# Book Review: The Cambridge Handbook of Corrective Feedback in Second Language Learning and Teaching

**DOI:** 10.3389/fpsyg.2021.725160

**Published:** 2021-08-02

**Authors:** Xueli Liu, Junyan Wei

**Affiliations:** ^1^School of Foreign Languages and Cultures, Nanjing Normal University, Nanjing, China; ^2^School of Foreign Language Teaching, Nanjing University of Chinese Medicine, Nanjing, China

**Keywords:** corrective feedback, second language, learning, teaching, methodological models, individual differences

Corrective feedback (CF) refers to any signal that a learner's utterance may be erroneous in some way and it is also known as “negative evidence” in the field of second language acquisition (SLA). Research on CF started from debates over its usefulness, and more recently extended to finding the most effective of CF methods and influencing factors. The book Corrective Feedback in Second Language Learning and Teaching, edited by Nassaji and Kartchava (2021), is a timely collection of the most influential studies on this topic.

In this book, all together 59 authors contributed 36 chapters, which are organized into 8 parts. Part I works as an introductory section, providing readers with sufficient background and the dichotomy between the behaviorist and innatest theories. Part II presents methodological approaches, in which readers can find not only commonly used tools to measure the effectiveness of CF, but also methods related to research synthesis. Part III marshals the delivery modes of CF, including three verbal modes (oral, written, technology-mediated) and one non-verbal mode (gesture). The next three parts explore several matters that impact CF effectiveness. For example, how do the provider, intensity, and timing of CF impact learning (Part IV)? Are there relationships between L2 skills and sensitivity to CF (Part V)? Does context impact the effectiveness CF (Part VI)? The last two parts discuss CF from both teachers' and learners' perspectives (Part VII) and call our attention to the importance of considering the specific individuals, tasks, and languages when interpreting results (Part VIII).

There are many things to be admired about this book, which is the first one to provide comprehensive research on CF. Firstly, the book systematically brings together 36 most cutting-edge studies of CF: theoretical and empirical studies perfectly complement each other, different language skills in various contexts are thoroughly examined, and both teachers' and learners' perspectives are highlighted. Many of the studies are inspirational, providing readers a plethora of possible topics for future research. For example, a current trend in the field is to include computer-mediated and mobile-technology-mediated contexts, especially as remote schooling gradually becomes the norm. For another, it is novel to include gestures as a delivery mode of CF. Most previous studies focus on verbal features of CF, and non-verbal features, such as gestures, are not given sufficient attention. The combination of verbal and non-verbal delivery modes is a promising topic for future SLA research. Although each of the 8 parts in this book have an independent topic, they coalesce to form clear story, enabling readers to have a systematic understanding on CF, from theory to practice. Therefore, this book is very reader-friendly and works perfectly as a handbook for scholars and teachers in the field of SLA.

Secondly, methodologically, the book not only introduces some tools to measure the effectiveness of CF, but also highlights the importance of research synthesis. Part II provides almost all tasks to examine the effectiveness of CF, including picture description task, consensus task, jigsaw task, direct and indirect test, think-aloud, immediate recall, stimulated recall, interview etc. These practical methods will help scholars in the field of SLA, but more importantly, they can be perfectly utilized to help foreign language teachers understand how CF works in L2 classrooms. In addition to evaluating tools of CF, another crucial methodological practice is also provided to the readers–research synthesis. To quote Dan Brown in Chapter 8, “the general aim of this book is to synthesize recent developments and current research on CF” (p. 164). Narrative literature review is a traditional approach to describe the current research situation; however, it is heavily reliant on the authors' personal judgements. Comparatively, research synthesis is a quantitative approach to combining data from existing studies to establish a complete summary on a particular topic. Two methods of research synthesis (meta-analysis and methodological synthesis) are introduced to readers. Research synthesis is commonly applied in the evidence-based medicine studies and gradually becomes popular in linguistic studies. It is heartening to see that this book highlights the importance of research synthesis, which we can apply it to SLA studies as well.

Thirdly, it is worth mentioning that this book also pays attention to individual differences, especially age and gender, which again inspires us to explore some possible topics for future CF research. For instance, individual differences in cognitive functions may also affect CF effectiveness. The relationship between CF and cognitive functions such as attention, working memory, learning motivation, and cognitive control can be further analyzed, and with the help of some online processing equipment (e.g., eye-tracking, ERP, and fMIR) and data analytical tools such as Linear Mixed Effects Modeling, this analysis could be even more promising. By understanding the impact of individual differences on CF, to quote the ancient philosopher Confucius, “teachers can better educate students according to their natural abilities.”

In conclusion, this book provides a comprehensive and systematic examination of CF in SLA. It not only provides methodological models for empirical research but also offers methods for research synthesis. It highlights the importance of both teachers and learners, and pays particular attention to learners' individual differences. The whole book is a valuable resource to researchers and teachers in the field of SLA due to its theoretical and practical significance. The publication of this book will broaden the arena of SLA research and even educational psychology.

## Author Contributions

XL: draft the structure of the review. JW: drafts and revision. Both authors contributed to the article and approved the submitted version.

## Conflict of Interest

The authors declare that the research was conducted in the absence of any commercial or financial relationships that could be construed as a potential conflict of interest.

## Publisher's Note

All claims expressed in this article are solely those of the authors and do not necessarily represent those of their affiliated organizations, or those of the publisher, the editors and the reviewers. Any product that may be evaluated in this article, or claim that may be made by its manufacturer, is not guaranteed or endorsed by the publisher.
